# When should undergraduate medical students do an intercalated BSc?

**DOI:** 10.3402/meo.v20.30599

**Published:** 2015-12-22

**Authors:** Omer A. Jamall, Sheeraz S Iqbal, Abeer Rizvi, Osman Nayeem, Shaan Rashid, Abul MH Khan

**Affiliations:** School of Medicine, Imperial College London, London, UK

Attaining an intercalated BSc (Bachelor of Science) during a medical degree is becoming increasingly common. Recent estimates indicate that over a third of all medical students complete an intercalated degree before graduating (1). A BSc can enhance one's academic portfolio through opportunities for publications and presentations (2). Furthermore, it can help students develop important skills for a career in medicine, including learning how to conduct research and critically appraise medical literature (3, 4). Studies demonstrate that those who complete intercalated BScs are more likely to score well in subsequent exams and secure their desired foundation year posts (5).

Intercalated BScs are typically done after the second, third or fourth year of the medicine degree. However, choosing when to intercalate can represent a dilemma for medical students and thus it is imperative to weigh up the pros and cons behind intercalating during the different stages of the medical degree.

An advantage of intercalating after the second year of education is that it allows a continuation of learning style. The first 2 years of medicine are ‘pre-clinical years’ and are traditionally taught by a lecture-based format. The course content for a BSc is taught in a similar vein and differs to the independent style of learning adopted during the third year of medical education. This is frequently more clinically based (as are the fourth and final years). Students may feel that they would benefit from not having to switch in and out of the contrasting learning methods. Additionally, an intercalated BSc can allow you to develop an appreciation for evidence-based medicine; students may find this beneficial before starting clinical placements.

Intercalating after third year is an option also chosen by many. This is popular amongst students as it can act to ‘break up’ clinical education. Furthermore, following the teaching received in third year, many of the students become equipped with the necessary skills to carry out more advanced clinical projects. Students who have also had a degree of clinical experience could perceivably be more likely to understand how findings from research can relate to and benefit patients.

We feel that it is important to consider these factors when deciding when to intercalate. We would recommend any student tailors their decision based on what aspects of the BSc they would find most beneficial. There is a wealth of evidence highlighting the benefits of completing an intercalated BSc (5); however, there are a lack of studies suggesting when it is most useful to do so. Ideally, more research needs to be done to assess the benefits and drawbacks of intercalating during the different years in medical school.

*Omer A. Jamall* School of Medicine Imperial College London London, UK Email: oj211@ic.ac.uk *Sheeraz S Iqbal* School of Medicine Imperial College London London, UK *Abeer Rizvi* School of Medicine Imperial College London London, UK *Osman Nayeem* School of Medicine Imperial College London London, UK *Shaan Rashid* School of Medicine Imperial College London London, UK *Abul MH Khan* School of Medicine Imperial College London London, UK
